# Prognostic model establishment and immune microenvironment analysis based on transcriptomic data of long-term survivors of pancreatic ductal adenocarcinoma

**DOI:** 10.1016/j.bbrep.2025.102280

**Published:** 2025-09-25

**Authors:** Lizhi Lin, Ragnar Norrsell, Roland Andersson, Xian Shen, Daniel Ansari

**Affiliations:** a Department of Surgery, Clinical Sciences Lund, Lund University, Skåne University Hospital, Lund, Sweden; b Department of Gastrointestinal Surgery, The Second Affiliated Hospital of Wenzhou Medical University, Wenzhou, China; c Department of Breast Surgery, The First Affiliated Hospital of Wenzhou Medical University, Wenzhou, China; d Department of Gastrointestinal Surgery, The First Affiliated Hospital of Wenzhou Medical University, Wenzhou, China

**Keywords:** Pancreatic cancer, Long-term survivors, Transcriptomics, Prognostic model, Tumor microenvironment, Drug sensitivity

## Abstract

Pancreatic cancer continues to be a major cause of cancer deaths worldwide. Characterizing the tumors of long-term survivors (≥5 years survival) would create opportunities in prognostic and therapeutic strategies. In this study, RNA sequencing data was used to identify differentially expressed genes (DEGs) in tumors of long-term survivors (LTS) vs short-term survivors (STS). Using LASSO-Cox regression, 4 prognostic DEGs, along with tumor stage, were utilized to develop a model for identifying high- and low-risk tumors. In Kaplan-Meier survival analysis, the high-risk group had significantly worse prognosis in both the training and validation cohorts. Using KEGG pathway gene signature sets, the high-risk group was found to have amplification of pathways, such as focal adhesion and ECM receptor interaction. The low-risk group, meanwhile, showed upregulation of specific metabolic pathways. Using ESTIMATE analysis, the high-risk group was found to have more stromal cell infiltration. Increased unpolarized macrophages and decreased inflammatory/anti-tumoral macrophages were also found in the high-risk group. Lastly, drug sensitivities were calculated and found to be generally higher in the high-risk group. This study reveals a model for predicting survival and drug sensitivity in pancreatic cancer. Genetic, molecular and tumor microenvironment characteristics of tumors from LTS and STS have been identified, highlighting opportunities for further research.

## Highlights

• The study of long-term survivors of pancreatic cancer may offer unique insights into tumor biology.
• We identified tumor gene signatures and immune microenvironmental features differentiating long- vs short-term survivors.
• We developed a gene-based risk score predicting prognosis and drug sensitivity.

## 1 Introduction

Pancreatic cancer remains one of the most challenging and deadly malignancies globally, responsible for approximately 467 000 deaths per year [1]. It has the lowest 5-year survival rate among all major cancer types [2]. The actual 5-year survival rate may be as low as 4.2 %, when considering only patients with a histopathologically verified ductal adenocarcinoma [3]. Surgery, when possible, and chemotherapy are current standards of care [4]. Targeted therapies are tested in attempts to improve prognosis, but have thus far failed in creating much needed clinical improvements [5].

The 5-year survival rate is commonly used as a measure of long-term survival. Characterizing those few who survive past this mark is of interest for prognostication, drug development and personalized treatment. This group has expectedly been shown to have lower stage and lower grade tumors [3]. A meta-analysis suggested predictors of long-term survivors (LTS) after resection may also include BMI, biomarker levels (e.g. CA19-9) and neutrophil-lymphocyte ratio [6]. The same study concluded that tumor-related factors (such as staging) have the strongest association with LTS. However, the underlying tumor qualities leading to differences in these factors and thus survival are not well understood.

One proposed mechanism for long-term survival is tumor expression of T-cell targetable neoantigens [7]. This may facilitate an effective immune response against the tumor, thereby promoting improved survival. The tumor immune microenvironment has also been recognized as a key factor, with tumors from LTS exhibiting higher levels of memory B cells and fewer M0 (unpolarized/non-activated) and M2 (pro-tumoral) macrophages [8]. Additionally, differences in the tumor microbiome have been observed between LTS and short-term survivors (STS, i.e., <5 years) [8,9].

Genetic analysis can provide a comprehensive understanding of the intrinsic tumor characteristics that influence survival. This study aims to identify differentially expressed genes (DEGs) between tumors from LTS and STS, using these genes to develop a model for identifying high- and low-risk tumors. The prognosis and characteristics of these groups will then be analyzed to better understand the factors driving pancreatic cancer aggressiveness and to explore potential therapeutic research avenues.

## 2 Materials and methods

### 2.1 Data collection and processing

Some 145 “Pancreas-Adenocarcinoma Ductal Type” RNA sequencing data samples with corresponding overall survival status and survival time were collected from The Cancer Genome Atlas (TCGA) – Pancreatic adenocarcinoma (PAAD) database (https://portal.gdc.cancer.gov/projects/TCGA-PAAD).

Some 200 RNA sequencing samples with complete survival data were obtained from Pancreatic Cancer Harmonized “Omics” analysis for Personalized Treatment (PACA-CA) in the International Cancer Genome Consortium (ICGC) database (https://platform.icgc-argo.org/). This dataset was labeled as “ICGC-CA” in our analysis. “Well differentiated” samples are labeled as “G1”; “Moderately differentiated” as “G2”; “Poorly differentiated”, “Undifferentiated”, and “Poorly differentiated to Undifferentiated” as “G3”. Four patients had more than 1000 “Not Available” (NA) expression values, and were therefore removed.

Some 68 “Pancreatic Ductal Adenocarcinoma” RNA sequencing samples with complete survival data were obtained from The Australian Pancreatic Genome Initiative (APIGI AU) (https://platform.icgc-argo.org/). This dataset was labeled as “ICGC-AU” in our analysis. “1 - Well differentiated” samples were labeled as “G1”; “2 - Moderately differentiated” as “G2”; “3 - Poorly differentiated” as “G3”. We identified an expression outlier sample (SA528676) from the ICGC-AU cohort based on PCA and distribution analyses. The sample was removed prior to batch effect correction to avoid distortion of the normalization model.

Some 165 RNA sequencing samples with complete survival data were obtained from GSE224564 datasets from DEO database (https://www.ncbi.nlm.nih.gov/geo/). The “Age” and “Gender” factors were missing in this dataset.

All RNA sequencing data were downloaded in the raw counts format.

A total of 49 RNA sequencing samples with complete survival data were obtained from the GSE78229 dataset, and 51 RNA sequencing samples with complete survival data were obtained from the GSE79668 dataset (https://www.ncbi.nlm.nih.gov/geo/), which were used for external validation in our analysis.

Patients who had survival time more than or equal to 1825 days (5 years) were defined as LTS; while those with survival time less than 1825 days (5 years) were defined as STS.

### 2.2 Datasets merging and normalization

The TCGA, PACA-CA, APIGI-AU and GSE224564 datasets had low expression genes filtered individually using the R function “filterByExpr”: the minimal count was set to 2, the minimal proportion was set to 20 %. The TMM normalization(Trimmed Mean of M-values)was applied to correct compositional bias using the R function “calcNormFactors”.

We obtained 16431 common genes and 573 samples among the 4 public databases (TCGA; PACA-CA; APIGI AU; GSE224564) before merging. For duplicated genes, the median was used as the gene value.

To correct for batch effects, we first identified the batch variable in the merged data. The sequencing depth was evaluated across all normalized datasets and the TCGA was chosen as the reference batch. We then created a model matrix (mod) that included group labels (LTS and STS) to account for potential biological variations between groups. The raw counts were Log2 transformed into log2-counts per million (log2-CPM) to stabilize variance before batch effect removal. R packages “sva” (3.54.0) and function “Combat” were used to remove the batch effect. The merged data were TMM normalized again after the batch effect removal using the calcNormFactors with default settings. Principal Component Analysis (PCA) was used to compare the data distribution before and after batch effect removal.

### 2.3 Differentially expressed genes analysis and LASSO-Cox analysis

DEG analysis between the LTS and STS groups was performed using the “edgeR” package (4.4.1). We considered the STS group as the control group and the LTS group as the experimental group. We constructed a design matrix (design) based on the group information. The dispersion of the data were estimated using the estimateDisp function. A generalized linear model was then fitted using glmQLFit, and hypothesis testing was performed with the glmQLFTest. Finally, the topTags function was used to extract and list the differentially expressed genes. Significant genes were defined as having a false discovery rate (FDR) < 0.05 and an absolute log (Fold change) (log (FC)) ≥1.

To adjust for tumor stage, we consider the combination of stage I and II as early stage, III and IV as late stage. In total, 529 samples had stage information. The “fastDummies” package (1.7.5) was used to convert the “AJCC III/IV” stage factor into a dummy factor. The stage dummy factor, DEGs, along with survival time and survival status for each sample were in the Least Absolute Shrinkage and Selection Operator (LASSO)-Cox regression. We used the “glmnet” R package (4.1.8) to identify genes associated with survival outcomes. A 10-fold cross-validation was used to evaluate model performance. The random seed (123) was set for reproducibility. The Lambda minimal ratio was set to 0.01. The optimal Lambda (Lamba.min) value was selected based on the lowest mean cross-validation error.

We constructed a Cox proportional hazards model initially including significant variables with coefficients >0.01 from LASSO-Cox analysis. Subsequently, a stepwise regression method (both forward and backward) was applied to select the most relevant variables based on the Akaike Information Criterion (AIC). From this model, we selected variables with p value < 0.1 for a more strict multivariable Cox model. Finally, we selected variables with p value < 0.05 and constructed the final prognostic model. The R function “ggforest” was used for the forest plot.

### 2.4 Survival analysis

The risk score of each patient was calculated using the LASSO-Cox prediction model. The “maxstat” R package (0.7.25) was used to calculate the best cut-off value for the risk score. All patients were divided into a “High risk” group or a “Low risk” group according to the cut off value. The best cut-off value was also applied to the external validation cohort. The “survival” (3.7.0) and “survminer” (0.5.0) R packages were used for Kaplan-Meier survival analysis. The “ggplot2” (3.5.1) was used to generate survival curves. The “timeROC” (0.4) package was used for Receiver Operating Characteristic Curve (ROC) and the area under the curve (AUC) according to the specificity and sensitivity.

### 2.5 GSVA and GSEA analysis

The “GSVA” R package was used for Gene Set Variation Analysis (GSVA). “c2.cp.kegg.symbols.gmt” was downloaded from the Molecular Signatures Database to calculate the enrichment score of each sample. The “limma” (3.62.1) package combined with the “eBayes” method was used with the GSVA scores to assess differential pathway activity between the “High risk” group and the “Low risk” group. Absolute log (FC) > 0.1 and Adjusted p-value <0.05 were considered to be significantly different. “clusterProfiler” (4.14.4) package was used for the Gene Set Enrichment Analysis (GSEA) analysis based on the log (FC) values. The log (FC) for each gene was calculated as log2(mean(expression high risk)) - log2(mean(expression low risk)). The “pheatmap” (1.0.12) package was used for visualization.

### 2.6 Immune analysis

The Estimation of STromal and Immune cells in MAlignant Tumor tissues using Expression data (ESTIMATE) scores of each sample were calculated using the “estimate” (1.0.13) R package. Using the precomputed stromal-related and immune cell-related gene signatures and our merged gene expression dataset, we obtained the “Stromal Score”, “Immune Score” and “ESTIMATE Score”.

The CIBERSORT algorithm was used to estimate the immune cellular composition in our merged dataset. Support vector regression (SVR) was employed to deconvolve and quantify the mixture of immune cell types and their proportions. The correlation between the CIBERSORT score and risk score of each sample was calculated using “spearman”.

### 2.7 Drug sensitivity analysis

The “oncoPredict” (1.2) R package was used to calculate the drug sensitivity of each sample in the merged dataset based on the gene expression. Drug sensitivity data, including gene expression profiles of pan-cancer cell lines and their IC50 values in response to multiple drugs, were obtained from the “Genomics of Drug Sensitivity in Cancer” database (https://www.cancerrxgene.org/). The drug sensitivity file was combined with the risk group data. The sensitivity difference between the two groups were analyzed using the Wilcoxon Rank-Sum Test. A p < 0.05 was consider as significant. The “ggboxplot” function was used for visualization.

## 3 Results

### 3.1 Clinical characteristics

To comprehensively analyze the genetic differences between LTS and STS of pancreatic adenocarcinoma, we downloaded the RNA sequencing data and corresponding clinical characteristics from TCGA-PAAD, PACA-CA, APIGI AU, and GSE224564 databases. Samples with incomplete survival data or more than 1000 missing (‘NA’) gene expression values were excluded. After normalization, one outlier sample (SA528676) was excluded. After merging these four public databases, we obtained a large dataset of 573 patients (Table 1). Among these patients, there were 17 LTS and 556 STS, resulting in a 5-year survival rate of 2.97 % in our analysis.

**Table 1:** Clinical characteristics of the merged training dataset.

	STS	LTS	Overall
	(N = 556)	(N = 17)	(N = 573)
**Age**			
Mean (SD)	65.0 (11.0)	66.9 (7.28)	65.1 (10.9)
Median [Min, Max]	66.0 [35.0, 87.0]	67.0 [54.0, 78.0]	66.0 [35.0, 87.0]
Missing	197 (35.4 %)	3 (17.6 %)	200 (34.9 %)
**Gender**			
Male	218 (39.2 %)	7 (41.2 %)	225 (39.3 %)
Female	175 (31.5 %)	7 (41.2 %)	182 (31.8 %)
Unknown	163 (29.3 %)	3 (17.6 %)	166 (29.0 %)
**Grade**			
G1	47 (8.5 %)	2 (11.8 %)	49 (8.6 %)
G2	267 (48.0 %)	5 (29.4 %)	272 (47.5 %)
G3	166 (29.9 %)	9 (52.9 %)	175 (30.5 %)
Unknown	76 (13.7 %)	1 (5.9 %)	77 (13.4 %)
**Stage**			
I	74 (13.3 %)	3 (17.6 %)	77 (13.4 %)
II	342 (61.5 %)	13 (76.5 %)	355 (62.0 %)
III	69 (12.4 %)	0 (0 %)	69 (12.0 %)
IV	27 (4.9 %)	0 (0 %)	27 (4.7 %)
Unknown	44 (7.9 %)	1 (5.9 %)	45 (7.9 %)

### 3.2 Identification of differentially expressed genes and development of a predictive model

**Fig. 1:**
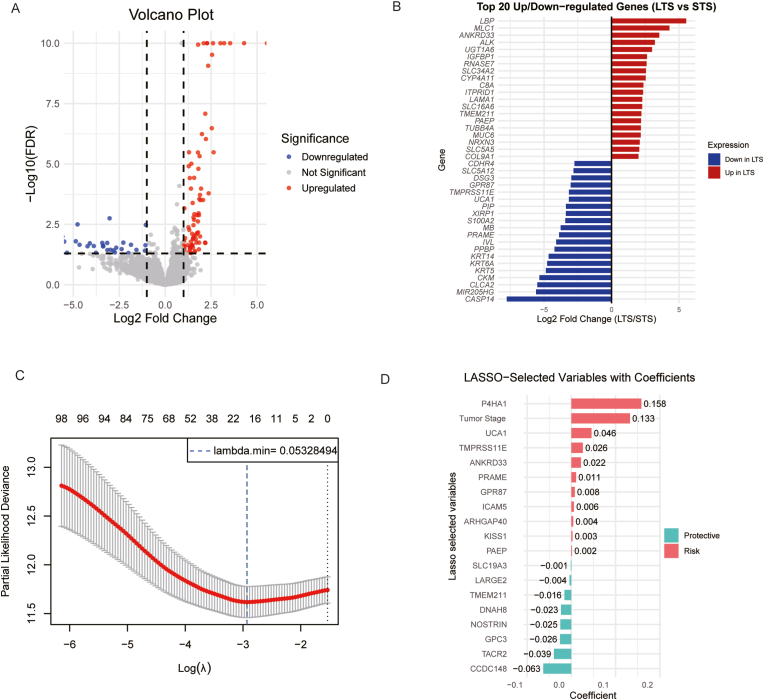
Differentially expressed genes between long-term survivors (LTS) and short-term survivors (STS) of pancreatic ductal adenocarcinoma: (A) Volcano plot of up- and downregulated genes in tumors of LTS compared to STS, (B) Bar plot of top 20 upregulated and top 20 downregulated genes, (C) LASSO-Cox plot with best Lamba value, (D) LASSO-Cox gene bar plot with selected genes.

A total of 108 DEGs were identified, of which 79 were upregulated in the LTS group, while 29 were downregulated (Fig. 1A). Fig. 1B shows the top 20 significantly expressed upregulated genes and downregulated genes.

**Fig. 2:**
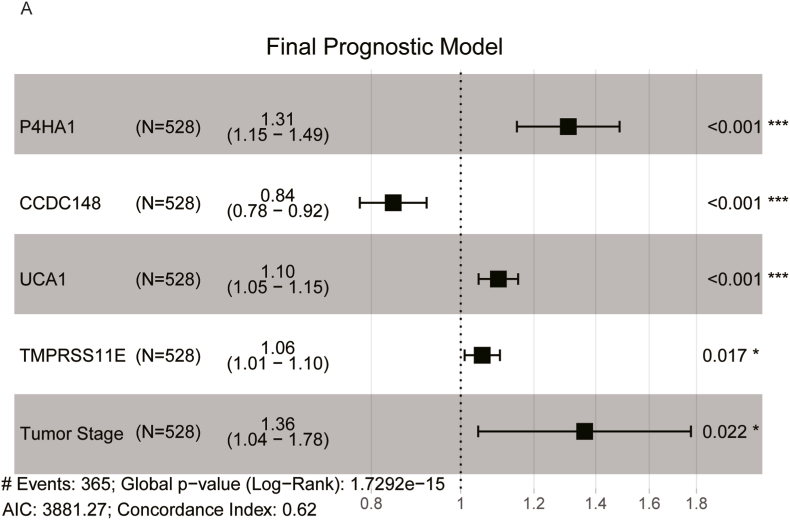
Forest plot illustrating the final prognostic model, which includes four genes alongside tumor stage.

By combining survival data with the expression of significant DEGs, we performed LASSO-Cox regression to identify prognostic DEGs differentiating the LTS from the STS. The optimal lambda value was determined to be 0.05328494 after 10-fold cross-validation (Fig. 1C), resulting in the selection of 19 genes with their corresponding coefficients (Fig. 1D). Based on the LASSO-Cox results, we initially constructed a Cox model incorporating variables with coefficients >0.1 from the LASSO-Cox analysis. A stepwise forward and backward regression method was then applied to select the most relevant variables based on the AIC. From this model, variables with a p-value <0.1 were chosen for a more stringent multivariable Cox model, and finally, variables with a p-value <0.05 were selected. The resulting risk score was calculated as follows:

Risk score = 0.268 ∗ P4HA1 - 0.168 ∗ CCDC148 + 0.094 ∗ UCA1 + 0.054 ∗ TMPRSS11E + 0.31 ∗ Tumor Stage (Fig. 2A).

### 3.3 Internal evaluation and external validation of the prediction model

**Fig. 3:**
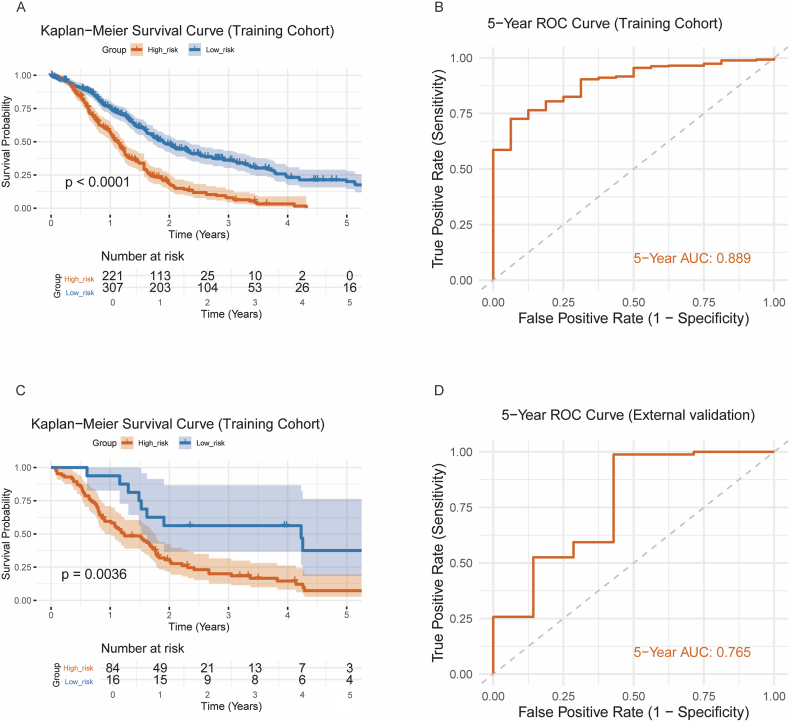
Kaplan-Meier survival curves and 5-year ROC curves for the merged dataset and the external validation cohort: (A) Comparison of overall survival between high- and low-risk tumors in the merged dataset. (B) AUC analysis at 5-years in the merged dataset. (C) Comparison of overall survival between high- and low-risk tumors in the external validation dataset. (D) AUC analysis at 5-years in the external validation cohort.

We calculated the risk score of each patient using the prediction model. The optimal cut-off value for the risk score (1.92) was determined using the “maxstat” R package. Patients with risk scores higher than the cut-off were classified into the high-risk group, while those with lower scores were classified into the low-risk group. Kaplan-Meier survival analysis showed a significantly reduced survival rate for the high-risk group in the merged dataset (p < 0.0001, Fig. 3A), with a hazard ratio (HR) of 2.35 (95 % CI 1.897–2.909). The area under the curve (AUC) was 0.889 for predicting 5-year survival (Fig. 3B), indicating good discriminatory ability.

For external validation, we merged the GSE78229 and GSE79668 datasets (Table 2). Based on the training model, we used the same cut off value of 1.92 for validation. The Kaplan-Meier survival analysis confirmed the worse survival in the high-risk group (p = 0.0036, Fig. 3C), with an HR of 2.732 (95 %CI: 1.351–5.524). The AUC was 0.765 for 5-year survival prediction (Fig. 3D).

**Table 2:** Clinical characteristics of the merged validation dataset.

	STS	LTS	Overall
	(N = 93)	(N = 7)	(N = 100)
**Age**			
Mean (SD)	64.8 (10.4)	55.3 (21.5)	64.0 (11.6)
Median [Min, Max]	67.0 [44.0, 85.0]	57.0 [28.0, 79.0]	66.0 [28.0, 85.0]
Missing	46 (49.5 %)	3 (42.9 %)	49 (49.0 %)
**Gender**			
Female	17 (18.3 %)	2 (28.6 %)	19 (19.0 %)
Male	30 (32.3 %)	2 (28.6 %)	32 (32.0 %)
Unknown	46 (49.5 %)	3 (42.9 %)	49 (49.0 %)
**Stage**			
I	11 (11.8 %)	2 (28.6 %)	13 (13.0 %)
II	76 (81.7 %)	4 (57.1 %)	80 (80.0 %)
III	5 (5.4 %)	1 (14.3 %)	6 (6.0 %)
IV	1 (1.1 %)	0 (0 %)	1 (1.0 %)
**Grade**			
G1	1 (1.1 %)	1 (14.3 %)	2 (2.0 %)
G2	23 (24.7 %)	1 (14.3 %)	24 (24.0 %)
G3	22 (23.7 %)	0 (0 %)	22 (22.0 %)
Unknown	47 (50.5 %)	5 (71.4 %)	52 (52.0 %)

### 3.4 Potential underlying pathways differentiating the high- and low-risk groups

To better understand the underlying differentially regulated pathways between the high- and low-risk groups, we performed GSVA and GSEA analysis.

**Fig. 4:**
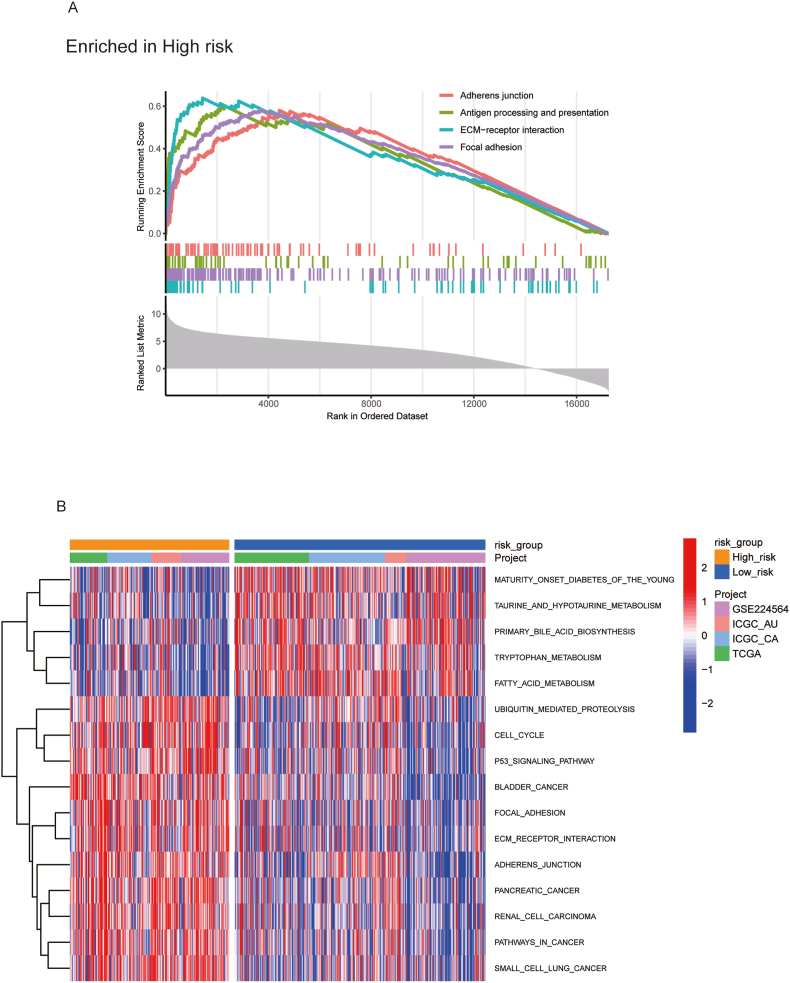
GSEA and GSVA analysis between high- and low risk groups: (A) The GSEA analysis based on the KEGG pathway database. (B) GSVA analysis based on the KEGG pathway database.

Using the gene expression matrix and the Kyoto Encyclopedia of Genes and Genomes (KEGG) pathway gene signature sets, we ranked genes based on the relative log fold change (log(FC)) between the two risk groups. The enrichment score was estimated accordingly. The result showed that adherens junction, ECM−receptor interaction, Focal adhesion and antigen processing and presentation pathways were significantly enriched in the high-risk group (Fig. 4A). Alterations in the extracellular receptor interaction, adherence junction and focal adhesion may facilitate cell adhesion, migration and invasion. The enrichment of antigen processing and presentation indicates dysregulation of the immune microenvironment in high-risk group patients, possibly related to immune escape and chronic inflammation. These findings suggest that patients in the high-risk group display more aggressive tumor features, which likely contribute to their poorer prognosis.

We also used the GSVA analysis to estimate enrichment score of each sample in the merged dataset. Differential pathway analysis between the high-risk and low-risk groups was conducted using linear models combined with eBayes. The results were visualized in a heatmap, highlighting potential upregulated and downregulated pathways in the two groups (Fig. 4B). Enrichment of the cell cycle pathway suggests abnormal cell cycle regulation in tumors. Consistent with the GSEA findings, pathways such as “FOCAL_ADHESION,” “ECM_RECEPTOR_INTERACTION,” and “PATHWAYS_IN_CANCER” were upregulated in the high-risk group. Additionally, other cancer-related pathways, including “PANCREATIC_CANCER,” “ADHERENS_JUNCTION,” and “P53_SIGNALING_PATHWAY,” were enriched, further emphasizing the aggressive tumor characteristics in this group.

Interestingly, in the low-risk group, upregulated pathways are primarily associated with metabolism, such as: “ALPHA_LINOLENIC_ACID_METABOLISM”, “GLYCINE_SERINE_AND_THERONINE_METABOLISM”, and “TRYPTOPHAN_METABOLISM”. The “MATURITY_ONSET_DIABETES_OF_THE_YOUNG” pathway, which comprises genes related to glucose metabolism, was also enriched in this group.

Combining the findings from GSVA and GSEA analyses, we propose that tumors in the high-risk group may have acquired more aggressive oncogenic functions, while those in the low-risk group exhibited favorable metabolic characteristics that may contribute to their better prognosis.

### 3.5 Tumor microenvironment difference between high- and low-risk groups

**Fig. 5:**
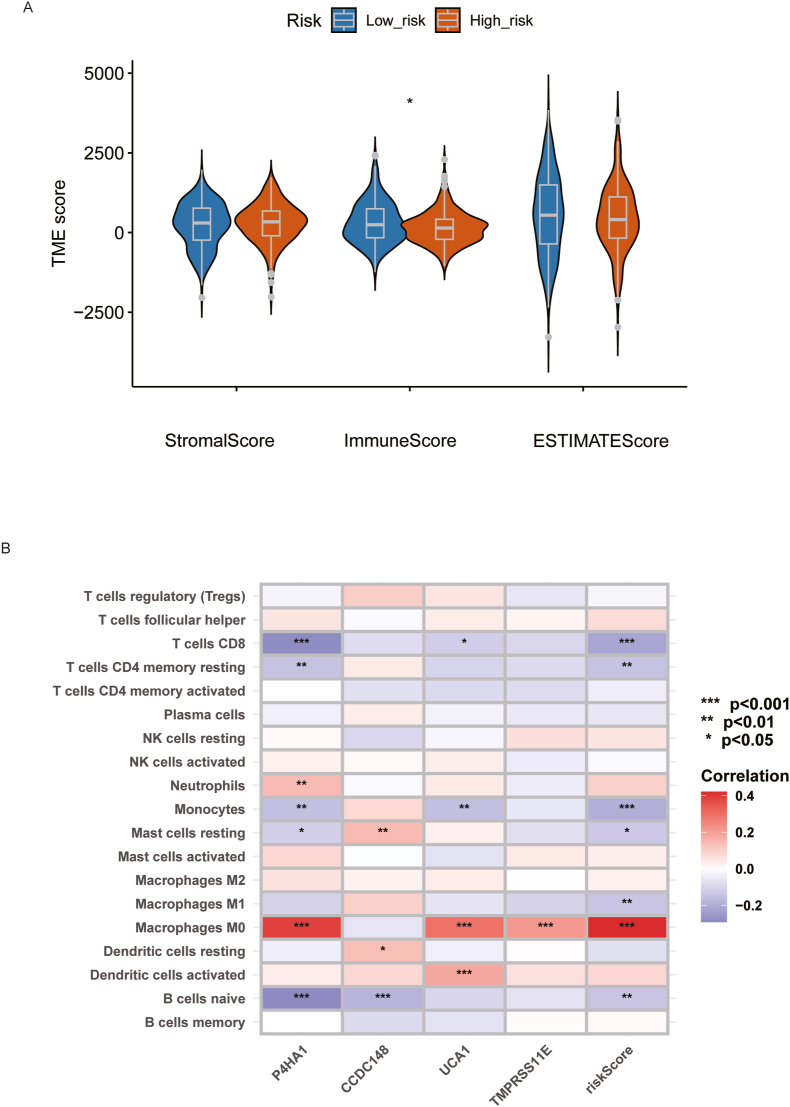
Tumor microenvironment analysis: (A) Violin plots of stromal score, immune score, and estimate score between high- and low risk groups, (B) Correlation between risk score and immune cells based on the CIBERSORT algorithm.

To thoroughly explore the biological characteristics of the two groups, we performed ESTIMATE analysis to assess stromal and immune cell infiltration levels. The violin plots revealed that the Immune Score in the high-risk group was significantly lower than in the low-risk group, while the Stromal Scores and ESTIMATE Scores showed no significant differences between the groups (Fig. 5A).

Although the Stromal Score did not differ between the two groups, suggesting overall proportion of non-tumor components and stromal cell infiltration was comparable, the heterogeneity of tumors and the complexity of the tumor immune microenvironment warranted further investigation. To this end, we conducted CIBERSORT analysis to evaluate the correlation between the risk score and specific immune cell components. Our analysis revealed a significant positive correlation between the risk score and M0 macrophages, and a negative correlation with M1 macrophages, monocytes, and CD8^+^ T cells and CD4 memory resting T cells (Fig. 5B).

M0 Macrophages represent unpolarized macrophages, whereas M1 macrophages, also known as classically activated macrophages, are crucial for combating acute inflammation and possess strong anti-tumor capabilities [10,11] These findings suggest that in the tumor microenvironment of high-risk patients, M0 macrophages may encounter challenges in polarizing into M1 macrophages with functional anti-tumor activity. This inability to transition could contribute to the more aggressive tumor characteristics observed in the high-risk group.

### 3.6 Clinical drug sensitivity analysis

To facilitate personalized clinical treatment using our predictive model and to identify potential novel therapeutic targets, we performed drug sensitivity analysis based on the merged gene expression data and risk group stratification.

**Fig. 6:**
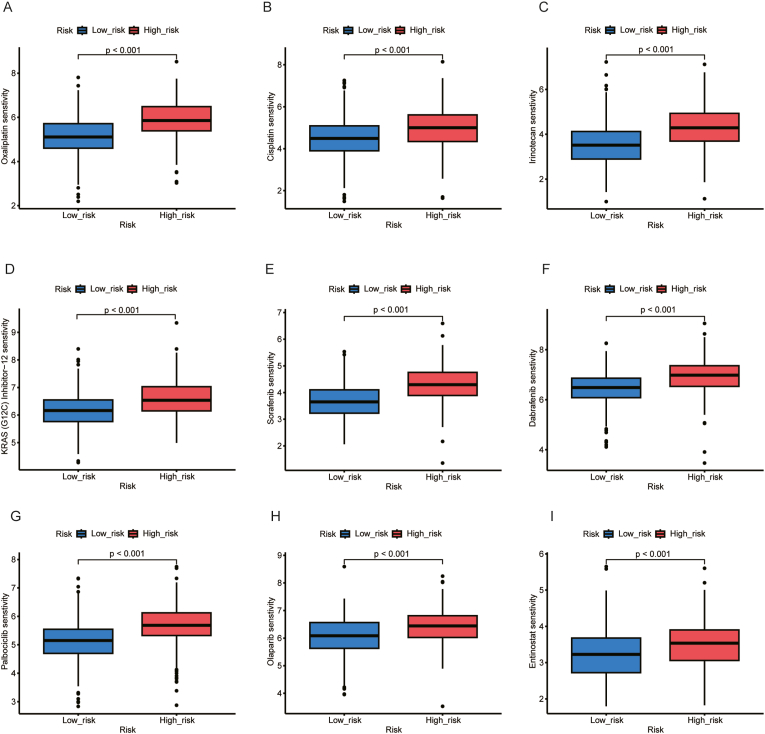
Drug sensitivity analysis between high- and low-risk groups: A) Oxaliplatin sensitivity, B) Cisplatin sensitivity, C) Irinotecan sensitivity, D) KRAS (G12C) Inhibitor −12 sensitivity, E) Sorafenib sensitivity, F) Dabrafenib sensitivity, G) Palbociclib sensitivity, H) Olaparib sensitivity, I) Entinostat sensitivity.

For chemotherapy, our results showed that the high-risk group had significantly higher sensitivity scores for Oxaliplatin (p < 0.001; Fig. 6A), Cisplatin (p < 0.001; Fig. 6B), and Irinotecan (p < 0.001; Fig. 6C).

The KRAS G12C mutation is detected in around 1–2 % of pancreatic cancer patients [12]. Our findings indicate that the high-risk group may have increased sensitivity to KRAS G12C inhibitors (p < 0.001; Fig. 6D).

Furthermore, the high-risk group demonstrated potential sensitivity to several targeted therapies, including, Sorafenib (a multikinase inhibitor; p < 0.001; Fig. 6E), Dabrafenib (a BRAF inhibitor; p < 0.001; Fig. 6F); Palbociclib (a CDK4/6 inhibitor; p < 0.001; Fig. 6G), Olaparib (a PARP inhibitor; p < 0.001; Fig. 6H), and Entinostat (an HDAC inhibitor; p < 0.001; Fig. 6I).

Overall, our analysis showed that the high-risk group patients may exhibit higher sensitivity to a range of chemotherapy and targeted drugs. These findings validate the clinical relevance of our predictive model for patient stratification and provide potential therapeutic insights for future clinical applications.

## 4 Discussion

In this study, we identified DEGs between LTS and STS of pancreatic cancer. From these DEGs, we selected 4 genes, along with tumor stage, to develop a prognostic model. The model's ability to accurately predict survival in an independent dataset supports the association of these genes with pancreatic tumor biology.

P4HA1 (Prolyl 4-hydroxylase subunit alpha 1) encodes a key enzyme involved in collagen biosynthesis, a process fundamental to maintaining the structural integrity of the extracellular matrix [13]. Beyond its structural role, P4HA1 plays a critical function in modulating the tumor microenvironment by influencing extracellular matrix remodeling, thereby facilitating cancer cell invasion and metastasis [14]. Recent studies have identified P4HA1 as an oncogene in pancreatic cancer, where it contributes to tumor progression and poor clinical outcomes [15,16].

UCA1 (urothelial carcinoma-associated 1), a long non-coding RNA, has been extensively studied for its oncogenic functions in pancreatic cancer [17]. UCA1 expression can be upregulated by oncogenic KRAS, a major driver mutation in pancreatic ductal adenocarcinoma [18]. Functionally, UCA1 contributes to tumorigenesis by modulating several key signaling pathways, including the Hippo pathway, and interacting with various microRNAs [[18], [19], [20]]. These interactions enhance cancer cell proliferation, invasion, and survival. Additionally, UCA1 has been implicated in the development of drug resistance, highlighting its potential as both a biomarker and a therapeutic target [21].

TMPRSS11E, also known as DESC1, is a type II transmembrane serine protease that has been implicated in cancer progression. In non-small cell lung cancer, TMPRSS11E promotes cellular proliferation and migration through a TMPRSS11E–PAR2–EGFR–STAT3 positive feedback loop, which amplifies oncogenic signaling [22]. Furthermore, in colorectal and melanoma cell lines, TMPRSS11E has been shown to activate the AKT pathway, enabling resistance to MEK1/2-ERK inhibition, thereby contributing to treatment resistance and sustained tumor growth [23]. However, the role of TMPRSS11E in pancreatic cancer remains unexplored. To date, no studies have comprehensively investigated its expression patterns, regulatory mechanisms, or functional contributions in pancreatic tumorigenesis. Elucidating the potential oncogenic role of TMPRSS11E in pancreatic cancer warrants further investigation and may uncover novel targets for therapeutic intervention.

The upregulation of specific metabolic pathways in the low-risk group is a particularly intriguing finding. The critical role of metabolic pathways in pancreatic cancer has been well established [24,25]. It is plausible to speculate that the reliance of low-risk tumors on these particular pathways may render them more susceptible than if they had utilized alternative mechanisms for energy production. Further investigation into this aspect could provide valuable insights.

Although we did not observe significant differences in the Stromal Score between the high- and low-risk groups, KEGG pathway analysis revealed enrichment of ECM-receptor interaction and adherens junction pathways. The increased activity of pathways such as adherens junction, focal adhesion, and ECM-receptor interaction in the high-risk group suggests heightened cell-matrix interactions within their tumor microenvironment compared to the low-risk group. ECM receptors, such as fibronectin receptors (e.g., the integrin family), laminin receptors, and collagen receptors, play a crucial role in tumor progression [26]. ECM remodeling and increased matrix stiffness contribute to malignant behaviors including invasion, migration, and angiogenesis through these receptors, ultimately leading to poorer prognosis [27,28]. Consistently, the upregulation of focal adhesion and adherens junction pathways may promote tumor growth and survival, as well as enhance anoikis resistance in tumor cells [29,30]. Thus, the risk score may partially capture the dysregulation of the tumor-stroma interface at the molecular level, adding another layer of biological relevance to the model.

Fewer M0 macrophages in the high-risk score patients is in line with previous research [8]. Combined with increased M1 (anti-tumoral) macrophages with the low-risk score, this implies that the activation of M0 macrophages into M1 is hindered in high-risk tumors, decreasing the ability of the immune system to combat the tumor. This naturally begs the question of whether increasing M1 polarization could be a therapeutic strategy. Indeed, macrophage immunotherapy has been discussed and researched with M1 polarization being possible through for example CD40 agonism [31]. A phase II trial including a CD40 agonist in pancreatic cancer unfortunately failed to show clear improvement [32], but with correct patient selection and the right drug combination it may be a viable strategy. The CIBERSORT analysis revealed that the risk score was negatively correlated with CD8^+^ T cells, and resting memory CD4^+^ T cells, which may impair effective immune surveillance [33,34], suggesting that patients with higher risk scores tend to have an immunosuppressive tumor microenvironment. Our prognostic model reflects the immunological landscape of the tumor, offering mechanistic insight into the observed survival differences. However, all these findings await experimental validation through techniques like immune cell profiling.

As mentioned, the drug sensitivity analysis indicates that this model may, with further analyses, be of use in deciding treatment regimens for patients. The higher general drug sensitivity in the high-risk group may be surprising since the patients had worse prognosis. The OncoPredict algorithm not only calculated the drug sensitivity based on specific gene expression but also related upstream or downstream pathways. It is possible that high risk patients had higher gene expression (e.g. KRAS G12C) or more active related gene pathways, which could be targeted by some of the therapies.

There are limitations in this study including the small number of LTS. This reduces the generalizability of the DEG analysis. A larger sample would be able to better validate the predictive ability of the model. Secondly, there was an underrepresentation of stage 4 tumors in the databases used. This is likely due to high stage-cancer not being resected and undergoing tumor analysis. Our prognostic model included clinical stage as a covariate in multivariate Cox regression analysis. This adjustment helps mitigate the potential confounding effect of stage distribution on survival prediction. Further external validation in more advanced-stage cohorts would be valuable to ensure the broad applicability of the model. It is possible that other DEGs would have been identified had this large group been included in the STS group. Lastly, the drug sensitivity analysis here is based on predicted sensitivity by OncoPredict. In vitro and clinical drug sensitivity comparisons are needed before this model could be used to decide a patient's treatment.

## 5 Conclusion

We have identified genes whose expression levels characterize the tumors of LTS of pancreatic cancer. Using these, we have created a risk score-model that can predict prognosis and drug sensitivity, hopefully leading to clinical utility. Our analysis of up- and downregulated pathways and tumor microenvironment properties identifies entry points for research into the mechanisms of pancreatic cancer aggressiveness. Further research should analyze the identified genes, as well as the metabolism and immune landscape of tumors of LTS to gain better understanding and identify therapeutic strategies.

## Ethical approval

The used data registries contain strictly de-identified patient data. The Swedish Ethical Review Authority approved the study protocol and waived the need for written informed consent from the participants.

## CRediT authorship contribution statement

Lizhi Lin: Writing – original draft, Visualization, Validation, Methodology, Investigation, Formal analysis, Data curation, Conceptualization. Ragnar Norrsell: Writing – review & editing, Methodology. Roland Andersson: Writing – review & editing, Supervision, Resources, Project administration. Xian Shen: Writing – review & editing, Methodology, Investigation. Daniel Ansari: Writing – review & editing, Supervision, Methodology.

## Funding

This work was supported by the 10.13039/501100002794Swedish Cancer Society, the 10.13039/501100004359Swedish Research Council, the Crafoord Foundation, the Ingrid and Sverker Persson Foundation, Regional Research Support and ALF funding from Region Skåne, as well as the Leading Talents in Scientific and Technological Innovation from Zhejiang Provincial Ten Thousand Talents Plan (Grant Numbers: 2019R52021, 2021R52024), the Key R&D Program of Zhejiang Province (Grant Numbers: 2020C03029, 2021C03120), the 10.13039/501100007194Wenzhou Municipal Science and Technology Bureau (Grant Number: ZY2022015), and the 10.13039/501100004543China Scholarship Council (Grant Number: 202208330338).

## Declaration of competing interest

The authors declare that they have no known competing financial interests or personal relationships that could have appeared to influence the work reported in this paper.

## Data Availability

Data will be made available on request.
